# Quantitative proteomics analysis of glioblastoma cell lines after lncRNA HULC silencing

**DOI:** 10.1038/s41598-021-92089-z

**Published:** 2021-06-15

**Authors:** Shan Ye, Jing Wu, Yiran Wang, Yuchen Hu, Tiantian Yin, Jie He

**Affiliations:** 1 Anhui Provincial Hospital Affiliated to Anhui Medical University, Hefei, China; 2 Department of Pathology, The First Affiliated Hospital of USTC, Hefei, China; 3 Department of Pathology, Anhui Provincial Cancer Hospital, Hefei, China

**Keywords:** Cancer, Cell biology, Biomarkers, Oncology

## Abstract

Glioblastoma multiforme (GBM) is a life-threatening brain tumor. This study aimed to identify potential targets of the long noncoding RNA (lncRNA) HULC that promoted the progression of GBM. Two U87 cell lines were constructed: HULC-siRNA and negative control (NC). Quantitative real-time PCR (qRT-PCR) was performed to validate the transfection efficiency of HULC silencing vector. Mass spectrometry (MS) was used to generate proteomic profiles for the two cell lines. Gene Ontology (GO) and Kyoto Encyclopedia of Genes and Genomes (KEGG) pathway enrichment analyses were performed to distinguish HULC-related genes and pathway mapping. Colony formation, Transwell, and wound-healing assays were used to investigate the functional effects of HULC knockdown on GBM. We identified 112 up-regulated proteins and 24 down-regulated proteins from a total of 4360 quantified proteins. GO enrichment illustrated that these proteins were mainly involved in organelle structure, catalysis, cell movement, and material metabolism. KEGG pathway analysis indicated that some of these proteins were significantly enriched in tight junction, metabolic pathways, and arachidonic acid metabolism. In vitro experiments demonstrated that HULC knockdown inhibited GBM cell proliferation, invasion, and migration. Our KEGG analyses revealed that PLA2G4A was a shared protein in several enriched pathways. HULC silencing significantly down-regulated the expression of PLA2G4A. Knockdown of HULC changed the proteomic characteristics of GBM and altered the behaviors of GBM cells. Specifically, we identified PLA2G4A as an HULC target in GBM. This study provides a new perspective on the mechanisms and potential drug targets of GBM treatment.

## Introduction

Glioblastoma multiforme (GBM) is the most common central nervous system (CNS) tumor in adults, characterized by a highly malignant aggressive behavior^1^. At present, the standard treatment for GBM includes surgery, radiotherapy, and chemotherapy since targeted therapy performs poorly. Although some new treatments have been developed, such as photodynamic therapy and immunotherapy^2,3^, their efficacy needs to be further evaluated. Disappointingly, even with treatment, GBM patients have a poor prognosis. According to a research in the United States, the 1-year survival rate of GBM patients is approximately 40.2%, and the 5-year survival rate is only 5.6%^4^. Therefore, there is an urgent need to investigate the underlying pathology of GBM to identify suitable biomarkers that can facilitate early detection and diagnosis, as well as further improve treatment and prognosis.

Long non-coding RNAs (lncRNAs) are described as a family of RNAs that are more than 200 nucleotides in length and harbor different functions according to their subcellular localization. LncRNAs are primarily involved in gene regulation through their interaction with other RNAs or proteins, including transcriptional regulation, post-transcriptional regulation, and epigenetic regulation^5^. Increasing evidence supports that lncRNAs play a vital role in tumorigenesis and progression.

The lncRNA HULC was first discovered in liver cancer tissues by Panzitt et al.^6^. It was later discovered that HULC could promote tumor growth as a lncRNA^7^. Several studies have also reported that HULC was highly expressed in other tumors, such as gastric cancer, colon cancer, and ovarian cancer. Investigation of the common underlying mechanisms of HULC in different cancers is ongoing^8–10^. Yan et al. suggested that over-expression of HULC might be utilized as a reference index for poor prognosis of GBM^11^. However, there are only a few studies that have focused on HULC’s mechanistic role in GBM^12^.

Proteomics has recently been widely used to identify new tumor biomarkers^13–15^. Mass spectrometry (MS) is the fastest growing, most dynamic, and promising technology in proteomics research. Liquid chromatography coupled with MS (LC–MS) is considered an effective tool in the discovery and verification of disease biomarkers due to its high sensitivity, precision, accuracy, and strong quantitative capability^16^.

In this study, we constructed stable HULC knockdown cell lines to verify the effect of HULC silencing in vitro. Combining highly sensitive quantitative technology with bioinformatics analysis, our study systematically identified differentially expressed proteins and discovered the potential mechanism by which the lncRNA HULC affects GBM tumor growth. Our results provide new insights into the targets involved in GBM pathogenesis, providing a theoretical basis for targeted GMB therapy.

## Results

### qRT-PCR detection of HULC expression in two stably-transfected cell lines

**Figure 1:**
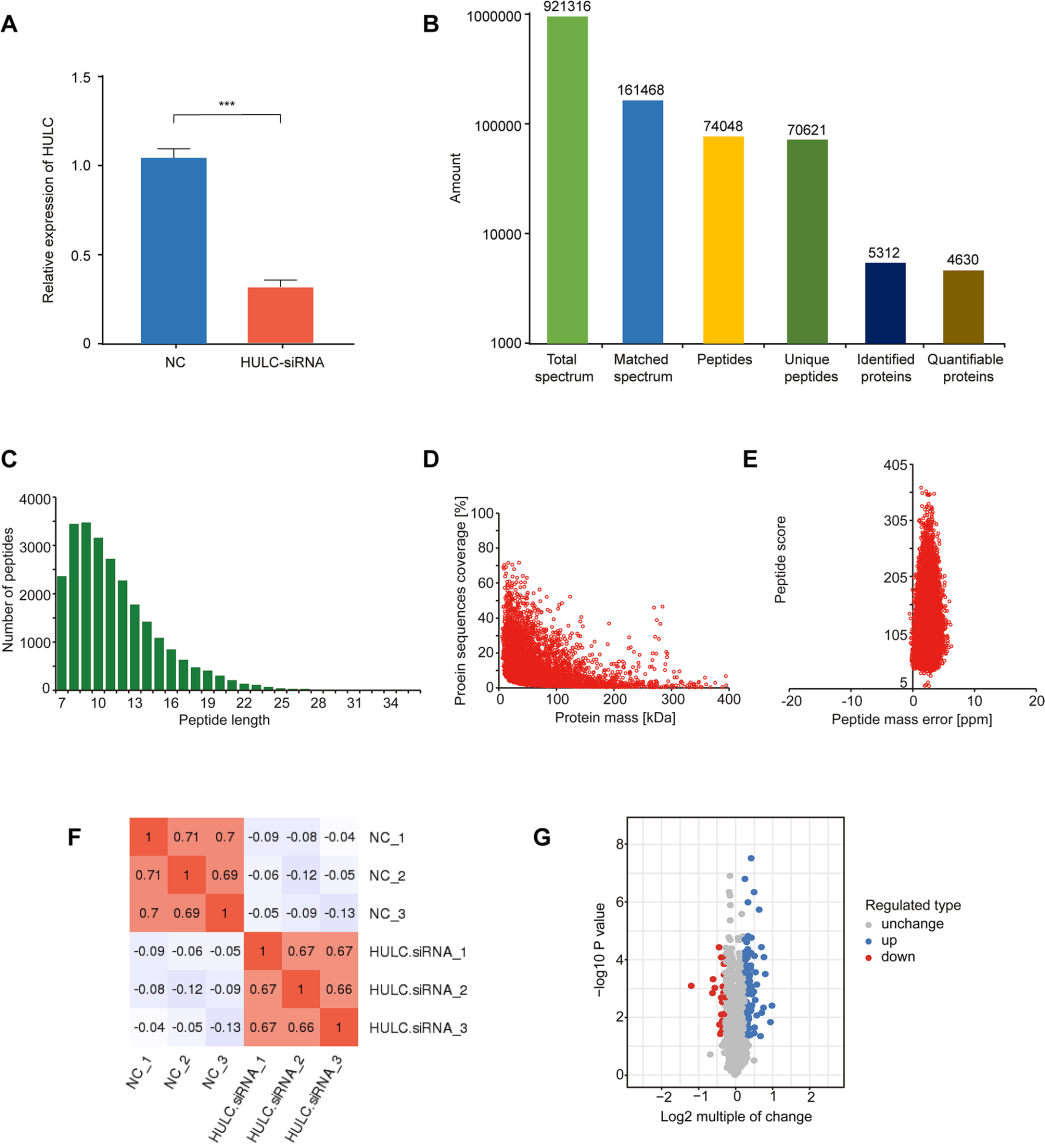
Proteins exhibiting altered expression in HULC-siRNA cells. **(A)** qRT-PCR analysis of the relative expression level of HULC from U87 HULC-siRNA stable cell lines and normal control (NC) (P = 0.0003). **(B)** Protein identification using MS with spectrum extraction. **(C)** Peptide distribution in MS analysis. **(D)** Relationship between proteome coverage and molecular weight. **(E)** Mass tolerance of most spectra. **(F)** Pearson correlation coefficient between every two replicates. **(G)** Volcano plot of differentially expressed proteins quantified using LC–MS/MS.

The relative expression of HULC in both HULC-siRNA and NC stable cell lines was determined using qRT-PCR analysis. HULC expression was significantly greater in the NC cell line (1.043 ± 0.052) compared to the HULC-siRNA cell line (0.310 ± 0.038) (t = 11.35, P = 0.0003) (Fig. 1A). This demonstrated that the HULC-siRNA construct effectively reduced HULC expression.

### MS data and quality control testing

The MS data are shown in Fig. 1B. The effective spectra were filtered through a database search. In total, 5312 proteins were identified, among which 4630 were quantified (Fig. 1B). To validate the quality of the MS data, we analyzed two quality parameters—peptide mass tolerance and peptide length. Most peptides were within the range of 7–20 amino acids in length, in line with the general rules after trypsin digestion and HCD fragmentation (Fig. 1C). The molecular weight of the proteins negatively correlated with the coverage (Fig. 1D). The mass tolerance of most of the spectra was within 10 ppm, which was consistent with the high-precision characteristics of Orbitrap MS (Fig. 1E). The Pearson correlation coefficient between every two replicates was greater than 0.6, and there was no correlation between different groups, indicating that the obtained protein samples maintained good reproducibility (Fig. 1F).

### Identification of proteins exhibiting altered expression in HULC-siRNA cells

We performed LC–MS/MS to identify proteins exhibiting altered expression in HULC-siRNA cells. Comparisons of the quantitative values of protein expression were made between the mean and standard error of the readings of the HULC-siRNA and NC cells. The data were filtered as statistically significant when the P value was < 0.05, and a fold change in protein expression > 1.2 was regarded as up-regulation. Conversely, a fold change in protein expression < 1/1.2 was regarded as down-regulation. A total of 112 up-regulated proteins and 24 down-regulated proteins was detected (Table 1). The top five up-regulated proteins were APOC3, CCDC146, MPZ, CRYAB, and RNF7, and the top five down-regulated proteins were CCDC159, SASH1, ANXA8L1, PLA2G4A, and CYP51A1. A volcano plot shows the log2 (fold change) as the abscissa, and -log10 of the P value as the ordinate (Fig. 1G). Our results indicate that HULC knockdown alters the protein profile of GBM cells, which likely contributed to tumor pathogenesis.

**Table 1:** An overview of 136 differentially expressed proteins identified in GBM cell lines.

Protein accession	Protein description	Gene name	MW [kDa]	siRNA/NC ratio	P value
P02656	Apolipoprotein C-III	APOC3	10.852	2	0.0039812
Q8IYE0	Coiled-coil domain-containing protein 146	CCDC146	112.81	1.946	0.0149
P25189	Myelin protein P0	MPZ	27.554	1.764	0.00031934
P02511	Alpha-crystallin B chain	CRYAB	20.159	1.721	0.00008267
Q9UBF6	RING-box protein 2	RNF7	12.683	1.706	0.0046752
Q8TAC9	Secretory carrier-associated membrane protein 5	SCAMP5	26.104	1.65	0.0070596
Q01995	Transgelin	TAGLN	22.611	1.642	0.000037074
Q6RFH5	WD repeat-containing protein 74	WDR74	42.441	1.613	0.045603
P09493	Tropomyosin alpha-1 chain	TPM1	32.708	1.57	1.8564E−06
Q8N3V7	Synaptopodin	SYNPO	99.462	1.506	0.00073988
Q6ZN11	Zinc finger protein 793	ZNF793	46.926	1.504	0.0087173
P02794	Ferritin heavy chain	FTH1	21.225	1.468	0.00027678
P04114	Apolipoprotein B-100	APOB	515.6	1.46	0.0025445
P30838	Aldehyde dehydrogenase, dimeric NADP-preferring	ALDH3A1	50.394	1.443	0.000075171
P27658	Collagen alpha-1(VIII) chain	COL8A1	73.363	1.443	0.022883
O75882	Attractin	ATRN	158.54	1.433	0.036083
P08670	Vimentin	VIM	53.651	1.431	4.5998E−07
P21589	5'-nucleotidase	NT5E	63.367	1.422	0.00130019
Q99715	Collagen alpha-1(XII) chain	COL12A1	333.14	1.376	0.00162044
P48681	Nestin	NES	177.44	1.374	0.0038995
Q16643	Drebrin	DBN1	71.428	1.361	1.74651E−05
Q96C19	EF-hand domain-containing protein D2	EFHD2	26.697	1.361	0.00135728
Q08945	FACT complex subunit SSRP1	SSRP1	81.074	1.357	0.0009648
Q14315	Filamin-C	FLNC	291.02	1.355	3.0598E−08
Q7Z406	Myosin-14	MYH14	227.87	1.353	0.000059659
Q14123	Calcium/calmodulin-dependent 3',5'-cyclic nucleotide phosphodiesterase 1C	PDE1C	80.759	1.351	0.005461
Q14011	Cold-inducible RNA-binding protein	CIRBP	18.648	1.344	0.000161097
Q9BX67	Junctional adhesion molecule C	JAM3	35.02	1.339	0.041317
O94875	Sorbin and SH3 domain-containing protein 2	SORBS2	124.11	1.339	0.0061629
Q9UKA9	Polypyrimidine tract-binding protein 2	PTBP2	57.49	1.339	0.000198578
Q96CT7	Coiled-coil domain-containing protein 124	CCDC124	25.835	1.333	0.0060834
Q8WWI5	Choline transporter-like protein 1	SLC44A1	73.301	1.328	0.00079616
Q32NB8	CDP-diacylglycerol–glycerol-3-phosphate 3-phosphatidyltransferase, mitochondrial	PGS1	62.73	1.328	0.040244
Q8IWT1	Sodium channel subunit beta-4	SCN4B	24.969	1.323	0.0058581
Q9H936	Mitochondrial glutamate carrier 1	SLC25A22	34.47	1.318	0.00056274
Q14978	Nucleolar and coiled-body phosphoprotein 1	NOLC1	73.602	1.314	0.0003646
Q92804	TATA-binding protein-associated factor 2 N	TAF15	61.829	1.305	0.024261
P07305	Histone H1.0	H1F0	20.863	1.304	0.00041633
O15075	Serine/threonine-protein kinase DCLK1	DCLK1	82.223	1.3	0.00035882
Q9NQS1	Cell death regulator Aven	AVEN	38.506	1.295	0.0117635
Q7RTV2	Glutathione S-transferase A5	GSTA5	25.722	1.294	0.043362
P29972	Aquaporin-1	AQP1	28.526	1.29	0.00029842
P49006	MARCKS-related protein	MARCKSL1	19.529	1.287	0.0096379
P35579	Myosin-9	MYH9	226.53	1.279	1.53911E−05
P12814	Alpha-actinin-1	ACTN1	103.06	1.276	1.0319E−06
O00159	Unconventional myosin-Ic	MYO1C	121.68	1.274	0.000059025
Q6NZI2	Caveolae-associated protein 1	CAVIN1	43.476	1.271	0.000043943
Q5M775	Cytospin-B	SPECC1	118.58	1.271	0.003664
Q96KR1	Zinc finger RNA-binding protein	ZFR	117.01	1.269	0.00098296
P11532	Dystrophin	DMD	426.74	1.267	0.00143623
O43707	Alpha-actinin-4	ACTN4	104.85	1.266	0.00023728
Q15018	BRISC complex subunit Abraxas 2	ABRAXAS2	46.9	1.266	0.043177
Q9NYF8	Bcl-2-associated transcription factor 1	BCLAF1	106.12	1.264	0.0164608
P62888	60S ribosomal protein L30	RPL30	12.784	1.264	0.000083727
P42262	Glutamate receptor 2	GRIA2	98.82	1.263	0.00022379
Q15233	Non-POU domain-containing octamer-binding protein	NONO	54.231	1.263	0.000075548
P00966	Argininosuccinate synthase	ASS1	46.53	1.263	0.0076647
Q9UHB6	LIM domain and actin-binding protein 1	LIMA1	85.225	1.259	0.0050829
O43281	Embryonal Fyn-associated substrate	EFS	58.815	1.258	0.042856
Q9BVA1	Tubulin beta-2B chain	TUBB2B	49.953	1.258	0.0052591
Q15052	Rho guanine nucleotide exchange factor 6	ARHGEF6	87.498	1.256	0.00157834
P53999	Activated RNA polymerase II transcriptional coactivator p15	SUB1	14.395	1.255	0.000184969
Q9H2L5	Ras association domain-containing protein 4	RASSF4	36.748	1.253	0.00019808
Q9Y4J8	Dystrobrevin alpha	DTNA	83.9	1.25	0.043597
Q8IWA4	Mitofusin-1	MFN1	84.159	1.25	0.0151353
Q6GYQ0	Ral GTPase-activating protein subunit alpha-1	RALGAPA1	229.83	1.248	0.0142205
Q9Y3E1	Hepatoma-derived growth factor-related protein 3	HDGFL3	22.619	1.248	0.040119
Q9Y2D5	A-kinase anchor protein 2	AKAP2	94.659	1.245	0.0149596
Q13557	Calcium/calmodulin-dependent protein kinase type II subunit delta	CAMK2D	56.369	1.245	0.000024929
Q6DN90	IQ motif and SEC7 domain-containing protein 1	IQSEC1	108.31	1.244	0.000162383
P39019	40S ribosomal protein S19	RPS19	16.06	1.242	0.000164125
Q01130	Serine/arginine-rich splicing factor 2	SRSF2	25.476	1.242	0.033157
Q9UPQ7	E3 ubiquitin-protein ligase PDZRN3	PDZRN3	119.6	1.239	0.000083308
Q96T51	RUN and FYVE domain-containing protein 1	RUFY1	79.817	1.239	0.00116479
P08138	Tumor necrosis factor receptor superfamily member 16	NGFR	45.183	1.238	0.0092784
Q05682	Caldesmon	CALD1	93.23	1.238	0.00008386
Q9P2K5	Myelin expression factor 2	MYEF2	64.121	1.238	0.0029401
Q92556	Engulfment and cell motility protein 1	ELMO1	83.829	1.238	0.0050598
Q9Y6R0	Numb-like protein	NUMBL	64.891	1.235	0.00064428
Q6WCQ1	Myosin phosphatase Rho-interacting protein	MPRIP	116.53	1.235	0.0161641
Q9BQ89	Protein FAM110A	FAM110A	31.27	1.233	0.03976
Q14938	Nuclear factor 1 X-type	NFIX	55.098	1.229	0.0089995
O75914	Serine/threonine-protein kinase PAK 3	PAK3	62.309	1.225	0.0048774
P16403	Histone H1.2	HIST1H1C	21.364	1.224	0.000096264
Q13509	Tubulin beta-3 chain	TUBB3	50.432	1.222	0.030999
Q6ICG6	Uncharacterized protein KIAA0930	KIAA0930	45.794	1.221	0.0044973
Q9Y3Y2	Chromatin target of PRMT1 protein	CHTOP	26.396	1.221	0.000080975
P50579	Methionine aminopeptidase 2	METAP2	52.891	1.221	0.000102665
Q9P2X3	Protein IMPACT	IMPACT	36.476	1.22	0.00085821
Q9Y2E5	Epididymis-specific alpha-mannosidase	MAN2B2	113.98	1.22	0.00092274
Q96L93	Kinesin-like protein KIF16B	KIF16B	152.01	1.22	0.035896
P09471	Guanine nucleotide-binding protein G(o) subunit alpha	GNAO1	40.05	1.217	0.000060744
Q8NCN5	Pyruvate dehydrogenase phosphatase regulatory subunit, mitochondrial	PDPR	99.363	1.215	0.038562
O60315	Zinc finger E-box-binding homeobox 2	ZEB2	136.45	1.215	0.00147829
P57723	Poly(rC)-binding protein 4	PCBP4	41.481	1.215	0.0119597
O15061	Synemin	SYNM	172.77	1.212	0.00047684
P19338	Nucleolin	NCL	76.613	1.212	0.000095076
Q8N684	Cleavage and polyadenylation specificity factor subunit 7	CPSF7	52.049	1.209	0.00056162
Q14195	Dihydropyrimidinase-related protein 3	DPYSL3	61.963	1.209	0.00055675
P45973	Chromobox protein homolog 5	CBX5	22.225	1.209	0.000020132
Q9UBS8	E3 ubiquitin-protein ligase RNF14	RNF14	53.837	1.209	0.00137693
P23246	Splicing factor, proline- and glutamine-rich	SFPQ	76.149	1.209	0.0037786
P62244	40S ribosomal protein S15a	RPS15A	14.839	1.208	0.0093783
P17480	Nucleolar transcription factor 1	UBTF	89.405	1.208	0.000055962
P54792	Putative segment polarity protein dishevelled homolog DVL1P1	DVL1P1	73.253	1.206	0.0079626
Q9Y5J5	Pleckstrin homology-like domain family A member 3	PHLDA3	13.891	1.206	0.0066224
O95319	CUGBP Elav-like family member 2	CELF2	54.284	1.205	0.0039437
P22626	Heterogeneous nuclear ribonucleoproteins A2/B1	HNRNPA2B1	37.429	1.205	1.5989E−07
Q86V81	THO complex subunit 4	ALYREF	26.888	1.205	0.0036446
Q8WV24	Pleckstrin homology-like domain family A member 1	PHLDA1	45.016	1.205	0.00028372
Q5VIR6	Vacuolar protein sorting-associated protein 53 homolog	VPS53	79.652	1.203	0.0177626
Q9Y2B9	cAMP-dependent protein kinase inhibitor gamma	PKIG	7.9104	1.202	0.025196
P61586	Transforming protein RhoA	RHOA	21.768	0.833	0.0020439
P06703	Protein S100-A6	S100A6	10.18	0.829	0.00169719
Q6NYC1	Bifunctional arginine demethylase and lysyl-hydroxylaseJMJD6	JMJD6	46.461	0.827	0.0197959
Q969H8	Myeloid-derived growth factor	MYDGF	18.795	0.824	0.000081223
P18827	Syndecan-1	SDC1	32.461	0.82	0.0032242
Q9H900	Protein zwilch homolog	ZWILCH	67.213	0.817	0.007761
P53602	Diphosphomevalonate decarboxylase	MVD	43.404	0.815	0.00033664
P48735	Isocitrate dehydrogenase [NADP], mitochondrial	IDH2	50.909	0.814	0.000142651
Q9BWD1	Acetyl-CoA acetyltransferase, cytosolic	ACAT2	41.35	0.808	0.00140305
Q9ULF5	Zinc transporter ZIP10	SLC39A10	94.131	0.797	0.00086387
Q9NZA1	Chloride intracellular channel protein 5	CLIC5	46.502	0.796	0.027481
Q9Y5U8	Mitochondrial pyruvate carrier 1	MPC1	12.347	0.79	0.0135035
P23219	Prostaglandin G/H synthase 1	PTGS1	68.686	0.789	0.0030041
O60218	Aldo–keto reductase family 1 member B10	AKR1B10	36.019	0.776	0.0080781
P13521	Secretogranin-2	SCG2	70.94	0.774	0.0021022
P07451	Carbonic anhydrase 3	CA3	29.557	0.774	0.000084276
P05109	Protein S100-A8	S100A8	10.834	0.769	0.034876
Q12872	Splicing factor, suppressor of white-apricot homolog	SFSWAP	104.82	0.762	0.038515
Q6ZMG9	Ceramide synthase 6	CERS6	44.889	0.749	0.0181562
Q16850	Lanosterol 14-alpha demethylase	CYP51A1	56.805	0.742	0.000037594
P47712	Cytosolic phospholipase A2	PLA2G4A	85.238	0.686	0.00096189
Q5VT79	Annexin A8-like protein 1	ANXA8L1	36.879	0.664	0.00048202
O94885	SAM and SH3 domain-containing protein 1	SASH1	136.65	0.657	0.00145894
P0C7I6	Coiled-coil domain-containing protein 159	CCDC159	33.695	0.441	0.00081975

*MW* molecular weight, *FACT* facilitates chromatin transactions, *SSRP* structure specific recognition protein, *DCLK* double cortin like kinase, *PRMT* protein arginine methyltransferase.

### Functional classification of identified proteins

**Figure 2:**
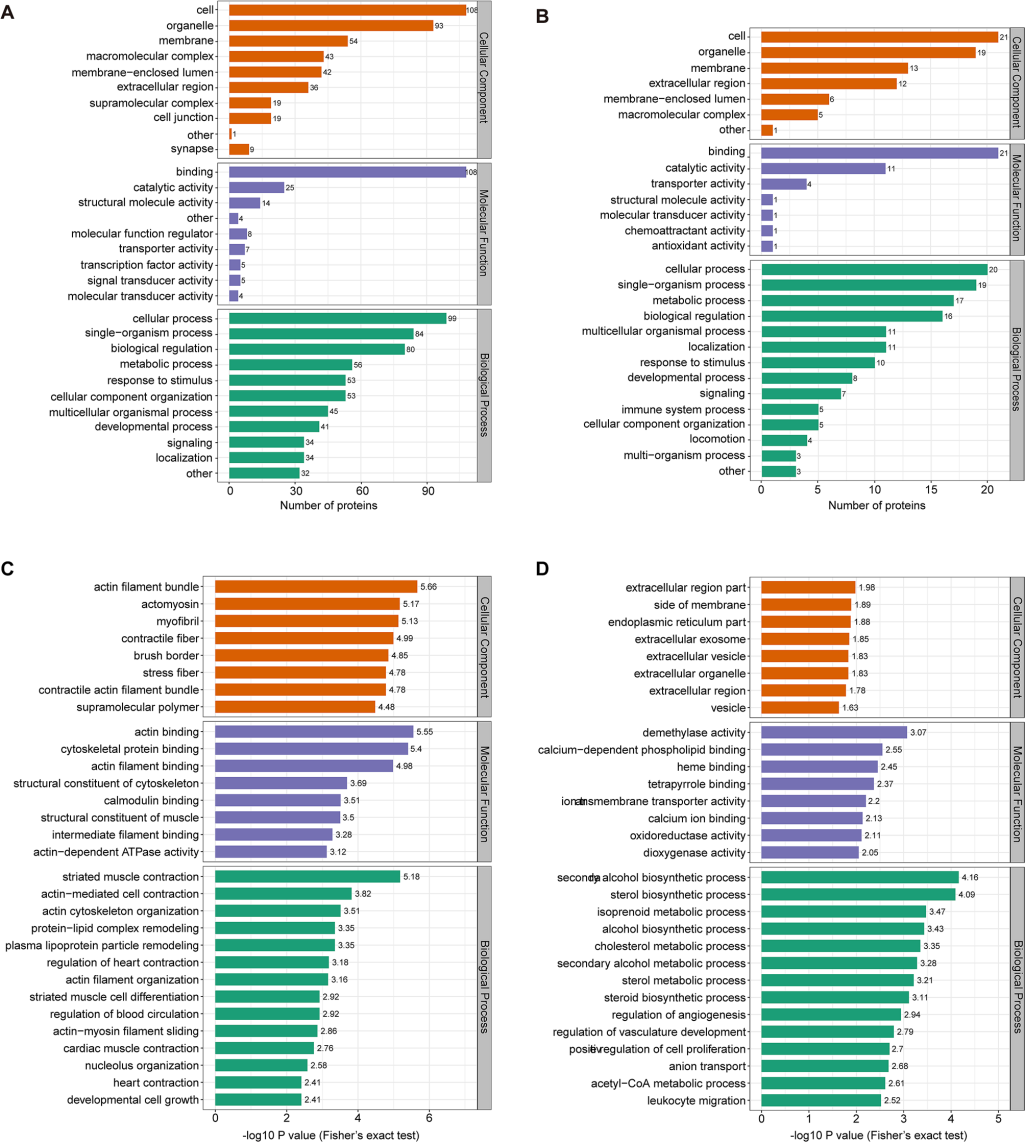
GO annotation and enrichment. **(A)** Selected proteins with a significant increase in abundance in cellular component, molecular function, and biological process, respectively. **(B)** Selected proteins with a significant decrease in abundance under the above classification. **(C)** GO enrichment analysis of the up-regulated proteins in biological process, cellular component, and molecular function. **(D)** GO enrichment analysis of the down-regulated proteins under the above classification.

To determine the functional characteristics of the identified proteins, three primary annotations were first obtained from the GO analysis: biological process, cellular component, and molecular function. In the GO secondary classification, the differentially expressed proteins were related to some important biological processes, including cells (87.5%), organelles (75.7%), and biological regulation processes (70.6%). These proteins participate in the composition of multiple cellular components (75.5%) and play a pivotal role in molecular binding (94.9%) and catalytic activity (26.5%). Moreover, this functional annotation appeared in both up-regulated and down-regulated proteins (Fig. 2A,B). The Fisher's exact test was further applied to the GO functional enrichment analysis of the identified proteins. As shown in Fig. 2C,D, when HULC was silenced, the proteins involved in the formation of the extracellular region were most significantly down-regulated, while proteins forming actin filament bundles were most obviously up-regulated. Demethylase activity and calcium-dependent phospholipid binding were significantly down-regulated, while proteins involved in actin binding were notably up-regulated. Moreover, various lipid metabolism pathways were significantly enriched in biological process (Fig. 2C,D). Directed acyclic graphs (Supplementary Fig. S1) not only intuitively reflect the enrichment differences of each GO classification, but also present the upper and lower hierarchical relationships of GO functions, indicating that GO function enrichment provided a deeper level of classification. For example, actin-dependent ATPase activity was significantly up-regulated at level 10 and calcium-dependent phospholipid binding was enriched in down-regulation at level 6.

### KEGG pathway annotation and enrichment

**Figure 3:**
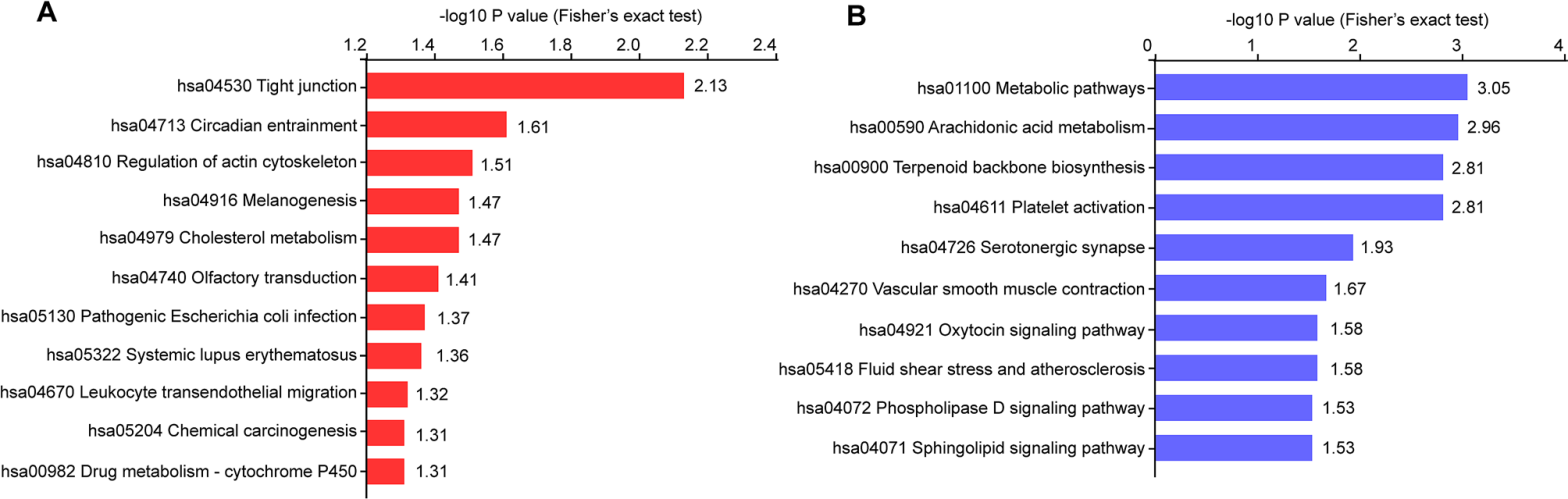
KEGG pathway enrichment in dysregulated proteins. **(A)** KEGG pathway enrichment of up-regulated proteins was performed using the Fisher's exact test, which indicates that tight junction was the most enriched pathway. **(B)** Plot exhibiting 10 down-regulated KEGG pathways with significant enrichment.

To understand the regulatory network associated with HULC knockdown, KEGG pathway analysis was performed with all differentially expressed proteins. We used the Fisher's exact test to further reveal the significantly enriched proteins in the annotated KEGG pathways. The P values are presented as -log10 conversion. Our results indicate that tight junction was the most enriched pathway and that there was a 3.4-fold up-regulation in this pathway following HULC knockdown. The down-regulated KEGG pathways were distributed in metabolic pathway, arachidonic acid metabolism, terpenoid backbone biosynthesis, and platelet activation (Fig. 3).

### Functional effects of HULC knockdown on U87 cells

**Figure 4:**
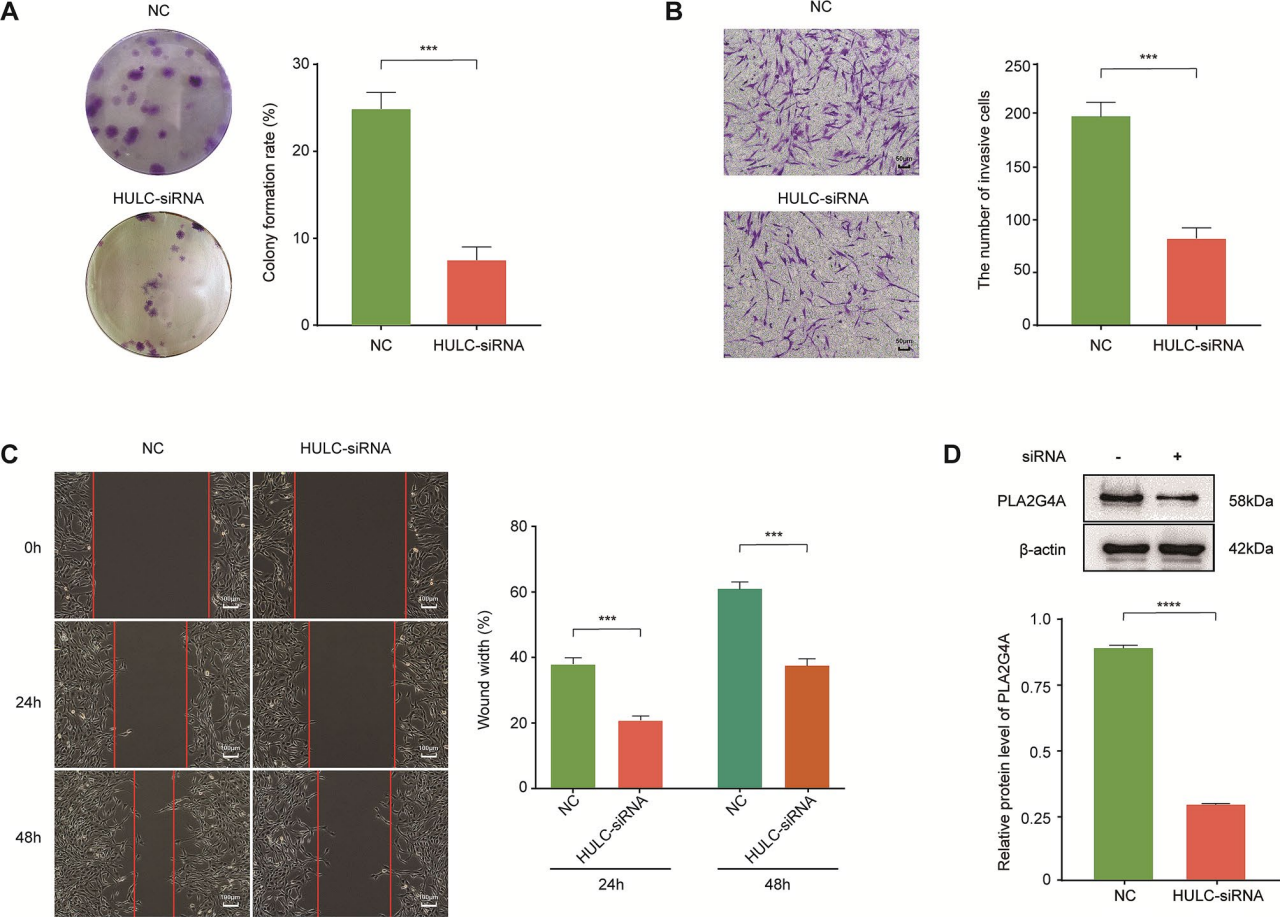
The effects of HULC inhibition to U87 cells. **(A)** Number of cell clusters reduced in response to HULC silencing (P = 0.0002). **(B)** HULC knockdown inhibited cell invasion determined using the Transwell assay (P = 0.0003). **(C)** Wound-healing assay was performed to elucidate cell migration after HULC knockdown (24 h, P = 0.0002; 48 h, P = 0.0003). **(D)** Protein abundance of PLA2G4A was decreased after HULC knockdown as determined by Western blot analysis (P < 0.0001). *P < 0.05, *P < 0.01, ***P < 0.001, ****P < 0.0001.

To assess the functional effects of HULC silencing on GBM cells, we first analyzed the effect of HULC knockdown on cell proliferation by colony formation assay in U87 cell lines. Proliferating colonies were scored as the 12 days after seeding. Compared to the negative control, the siRNA-mediated knockdown of HULC showed a 3.39-fold decrease in the number of clusters (P = 0.0002), indicating that cell proliferation was significantly inhibited (Fig. 4A). We next used the Transwell assay to determine whether HULC knockdown affected cell invasion. We found that HULC knockdown decreased cell invasion capability by 2.45-fold (P = 0.0003) compared to the negative control (Fig. 4B). The wound-healing assay showed that cell migration was also suppressed following HULC knockdown. Migration was reduced by 1.84-fold at 24 h (P = 0.0002), and 1.62-fold at 48 h (P = 0.0003) (Fig. 4C). These data indicate that HULC promotes GBM cell proliferation, invasion, and mobility in vitro.

### HULC regulates PLA2G4A protein expression

To identify key proteins regulated by HULC, we analyzed the common proteins of several significantly different signaling pathways (arachidonic acid metabolism, platelet activation, etc.). As a result, we found that the protein encoded by *PLA2G4A* plays a pivotal role in these pathways. Therefore, we used Western blot analysis to verify differences in PLA2G4A protein expression. Our results showed that knockdown of HULC significantly reduced the protein abundance of PLA2G4A (Fig. 4D).

## Discussion

GBM is a grade IV glioma and is the most aggressive malignant type of brain tumor. Increasing evidence demonstrates that many lncRNAs play various roles in a series of biological processes associated with the occurrence and development of GBM. For example, high expression of PVT1 in the nucleus can accelerate glioma cell proliferation, invasion, and aerobic glycolysis by inhibiting the expression of miR-140-5p^17^. GAS5-AS1 is another lncRNA expressed in glioma tissues. One study showed that GAS5-AS1 binded to miR-106b-5p to promote expression of downstream genes that play a role in inhibiting cell proliferation, migration, and invasion of glioma cells^18^. Emerging studies have invested the mechanisms by which lncRNAs influence other tumor behavior^19,20^. Much of these efforts have been focused on identifying highly specific and sensitive biomarkers to promote early diagnosis, predict prognosis, and provide potential therapeutic targets for different cancers.

The lncRNA HULC has been shown to be highly expressed in GBM cells compared to normal cells, as well as to promote the proliferation of GBM cells in vitro^11^. Zhu et al. found that silencing HULC inhibited glioma angiogenesis through the ESM-1-mediated PI3K/AKT/mTOR signaling pathway, resulting in GBM growth suppression^12^. However, the molecular mechanisms responsible for HULC’s regulation in GBM tumorigenesis have only begun to be scrutinized. Our study provides insight into this mechanism by identifying the potential targets of HULC in glioma cells.

Proteomics research has gained much attention in tumor biology studies. Farhadul et al. analyzed differences in the total proteome between esophageal squamous cell carcinoma and non-tumor cells using label-free shotgun proteomics combined with MS^13^. Zhao et al. screened tumor-specific antigens for high-grade serous ovarian cancer with MS, and found potential targets for ovarian cancer immunotherapy^14^. In this study, we obtained the proteome of HULC deficient GBM cells using TMT labeling. Through HPLC fractionation technology and LC–MS/MS analysis, we have new insights into some promising GBM biomarkers.

Among the differentially expressed proteins, we selected the top 5 up-regulated and 5 down-regulated proteins to further analyze. Based on a search of the PubMed database, none of these 10 proteins was previously reported to be related to HULC. Only two of the proteins, CRYAB and SASH1, have been studied in glioma^21,22^. Kore et al. demonstrated that the expression level of CRYAB was elevated in U373 glioma cells^21^. Methylation of SASH1 gene has been shown to inhibit cell adhesion and promote migration of astrocytes^22^. The remaining 8 proteins have not been previously reported to have any association with glioma. Although we did not further analyze these 8 proteins in the current study, we believe future in-depth analysis of these proteins will be helpful to better understand the underlying molecular mechanisms in GBM.

However, the GO findings were unexpected in that we identified some up-regulated proteins in cell activity, such as actin filament bundles and actin binding after HULC knockdown, that indicate that HULC suppression can promote tumor migration and invasion, which contradicts our functional results. We speculate that this discrepancy correlates with the complex characteristics of glial cells. In addition to participating in the formation of actin frameworks, glial cells can contract and phagocytose cell fragments, as well as repair and replenish neurons. We also acknowledge that there are likely differences between the MS data and actual verification results^23^. The biosynthesis pathway of the terpenoid backbone was significantly down-regulated, indicating that HULC knockdown exhibited a suppressive effect on cell proliferation. In addition, the strong down-regulation of the platelet activation pathway suggested that HULC was associated with GBM complications, such as thrombosis, to a certain extent^24^. DNA methylation is known to be an early event of tumorigenesis. MGMT (O6-methylguanine-DNA methyltransferase) is a DNA repair enzyme. It was reported that the methylation of the MGMT gene promoter is associated with glioma prognosis and recurrence^25^. Our proteomics analysis demonstrated that demethylase activity was decreased after HULC knockdown. Previous studies have also illustrated that demethylation behavior could promote tumorigenesis and progression^26,27^. Thus, the methylation or demethylation of HULC’s target gene should be investigated in future studies.

We found that the PLA2G4A encoded protein appeared in several notable KEGG pathways. Therefore, we hypothesized that PLA2G4A might be a potential downstream target of HULC. PLA2G4A is the most abundant subtype in the family of phospholipase A2. Phospholipase hydrolyzes membrane phospholipids and releases arachidonic acid, which is further involved in many pathophysiological processes, including inflammation, signal transmission, and cell growth^28^. Although one study showed that reducing PLA2G4A expression could promote the migration and invasion of esophageal squamous cell carcinoma^29^, others proposed that PLA2G4A was an oncogene^30–32^. For example, PLA2G4A has been shown to facilitate the metastasis of osteosarcoma by promoting epithelial-mesenchymal transition (EMT)^32^. Our proteomics data supported PLA2G4A’s role as an oncogene. Our independent Western blot assay also confirmed that the HULC knockdown significantly reduced PLA2G4A protein expression, suggesting that PLA2G4A might be a key protein that was regulated by HULC in GBM. Our enrichment analysis showed that PLA2G4A was involved in many important processes, including positive regulation of cell proliferation, calcium-dependent phospholipid binding, and the arachidonic acid metabolism pathway. Since the concept of tumor-promoting inflammation was proposed in 2011, tumor-associated inflammation has been considered a trigger point for cancer progression^33^. We hypothesize that PLA2G4A may also play an important role in the formation of tumor-related inflammation. Thus, targeting PLA2G4A might provide a promising therapy to GBM. Moreover, Tsuji S, et al. put forward that temozolomide might affect cPLA2^34^, which inspired us that targeting PLA2G4A might reverse temozolomide resistance.

This study has some limitations that should be noted. As we were limited to studying HULC with one cell line, and the validation of the LC–MS/MS data was performed only with one down-regulated protein. It remains for future experiments to further confirm the proteomic analysis results and to determine whether additional targets of HULC can be identified.

## Conclusions

In the era of big data, it is important to identify molecules that can guide the direction of disease research through in-depth analysis of gene and protein profiles. Our study indicates that HULC significantly changes the proteomic characteristics of U87 cell line, and that PLA2G4A is negatively regulated by HULC knockdown in GBM cells. This study provides a new perspective on the pathogenesis of GBM, and also provides a potential target for GBM treatment.

## Methods

### Cell culture and transfection

The human GBM glioma cell line, U87, was obtained from China Center for Type Culture Collection (Wuhan, China) and maintained in Dulbecco’s modified eagle’s medium (DMEM) (BD, USA) supplemented with 10% fetal bovine serum (FBS) (BD, USA). The cells were grown at 37 °C in a 5% CO_2_ atmosphere.

To generate lentivirus stable cell lines, cells were digested, resuspended, and plated in six-well dishes (Nest, China) at a density of approximately 10 × 10^5^ cells per well, and then cultured under the same conditions for 24 h. The lentiviral vectors (LV3-shNC and LV3-shHULC) and lentiviral packaging were purchased from GenePharma (Shanghai, China). The overall transfection procedure was in accordance with the recommendations of the manufacturer. A 200 μl lentivirus stock solution was diluted 5 times with DMEM containing 10% FBS according to the manufacture’s protocol. Infection enhancer polybrene (Sigma, USA) was added to a final concentration of 5 μg/ml. Stably-transfected cells were selected by puromycin (1 μg/ml, Sangon, Shanghai, China) and the green fluorescent protein (GFP) was observed under a fluorescence microscope (Olympus, Japan). After a 96 h in culture, the cells were harvested and stored at − 80 °C for subsequent experiments. Thus, two stable siRNA expressing cell lines were constructed, including HULC-siRNA and the negative control (NC). The sequence of shRNA targeting HULC was 5′-GAACTCTGATCGTGGACATTT-3′.

### Quantitative Real-Time Polymerase Chain Reaction (qRT-PCR)

In a week, RNA was extracted from two samples using a total RNA extraction kit (QIAGEN, Germany). cDNA was synthesized according to the protocol of the high-throughput cDNA reverse transcription kit (Thermo Fisher Scientific, USA). PCR was carried out under 40 cycles of 95 °C for 12 s, 55 °C for 30 s, and 72 °C for 1 min. The relative expression levels of the target genes were obtained using two variations of the 2^−ΔΔCt^ method. Each sample type was run in triplicate. Data were analyzed using the two-tailed t test. ACTB was used as the reference gene. The primers were as follows: HULC forward primer for the upstream sequence: 5′-TCAACCTCCAGAACTGTGATCC-3′, HULC reverse primer for the downstream sequence: 5′-TGCTTGATGCTTTGGTCTGTT-3′; ACTB forward primer for the upstream sequence: 5′-CGTGGACATCCGCAAAGA-3′, ACTB reverse primer for the downstream sequence: 5′-GAAGGTGGACAGCGAGGC-3′.

### Protein extraction

Cells were submitted to protein extraction after 3 months post transfection. Samples were sonicated 4 times on ice with a high intensity ultrasonic processor (Scientz, China) at 30% amplitude for no more than 7 consecutive seconds in diluted lysis buffer [8 M urea (Sigma, UAS), 1% protease inhibitor (Calbiochem, Germany), and 2 mM EDTA (Sigma, USA)]. The supernatant was collected by centrifugation at 12,000×*g* at 4 °C for 10 min. The protein concentration was determined using a BCA kit (Beyotime, China) according to the manufacturer’s instructions.

### Trypsin digestion

Dithiothreitol (DTT) (Sigma, USA) (5 nM) was used for every 0.3 mg protein reduction for 30 min at 56 °C. Iodoacetamide (Sigma, USA) was added to a final concentration of 11 nM, and the mixture was incubated at room temperature for 15 min in the dark. Trypsin (Promega, USA) was added using a trypsin/protein ratio of 1:50 for the first digestion overnight at 37 °C and trypsin/protein ratio of 1:100 for a second digestion for 4 h.

### Tandem mass tags (TMT) labeling

The digested peptides were desalted using a Strata X C18 SPE column (Phenomenex) and freeze-dried in the vacuum environment. Peptides were reconstituted in 0.5 M NH_4_HCO_3_ (Sigma, USA) and labeled using a TMT kit (Thermo Fisher Scientific, USA) according to the manufacturer’s protocol.

### High performance liquid chromatography (HPLC) fractionation

The Agilent 300 Extend C18 reversed-phase column (5 μm particles, 4.6 mm inner diameter, 250 mm length) was used to fractionate 0.2 mg peptides into 60 fractions with a gradient of 8% to 32% acetonitrile (Fisher Chemical, USA) under the condition of pH 9 over 60 min. The peptides were then combined into 9 components and freeze-dried by vacuum centrifuging.

### LC–MS/MS analysis

Two types of liquid chromatography mobile phases were first prepared. Phase A: an aqueous solution containing 0.1% formic acid (Fluka, USA) and 2% acetonitrile; Phase B: an aqueous solution containing 0.1% formic acid and 90% acetonitrile. Peptides were dissolved in phase A and separated using the EASY-nLC 1000 UPLC system (Thermo Fisher Scientific, USA) at a constant flow rate of 400 nL/min. The separation gradient was set to increase from 8 to 16% in phase B within 30 min, then increased to 30% within 25 min and 80% within 2 min, which was maintained for 3 min. The peptides were injected into the nanospray ionization source for ionization at a voltage of 2.0 kV. The precursor ions and the secondary fragments of the peptides were detected and analyzed using the Orbitrap Fusion Lumos high-resolution mass spectrometer (Thermo Fisher Scientific, USA). According to the data dependent acquisition (DDA) mode, the precursor ions with top 20 signal intensities after primary scan were fragmented with 32% fragmentation energy in the HCD collision cell. The secondary MS/MS scan then followed. The MS scan parameters are shown in Table 2.

**Table 2:** MS scan parameters.

Parameter	Value
**Range**	
Primary MS	350–1550 m/z
MS/MS	100 m/z (fixed starting point)
**Resolution**	
Primary MS	60,000
MS/MS	15,000
Automatic gain control	50,000
Signal threshold	50,000 ions/s
Maximum injection time	70 ms
Dynamically exclude time	30 s

### Bioinformatics analysis

The secondary MS data obtained was retrieved using the Maxquant database (v.1.5.2.8, http://www.maxquant.org/), and the relevant parameters are shown in Table 3.

**Table 3:** Relevant parameters for Maxquant database searching.

Parameter	Value
Protein database	SwissProt Human (20317 sequences)
Cleavage enzyme	Trypsin/P
Missing cleavages	2
Minimum length of peptide	7 amino acid residues
Maximum modifications of peptide	5
**Mass tolerance for precursor ions**	
First search	20 ppm20 ppm
Main search	5 ppm
Mass tolerance for fragment ions	0.02 Da
Fixed modification	Carbamidomethyl on Cys
Variable modification	oxidation on Met, N-terminal acetylation
Quantitative method	TMT-6plex
FDR for protein identification	1%
FDR for PSM identification	1%

The quantitative values of each sample in three replicates were obtained. The Pearson correlation coefficient was calculated between two pairs to assess whether the results of replicate samples were statistically consistent. Using the average of the three quantitative values, we calculated the ratio of the average between the two samples. Fold change was defined as the ratio of the average values of HULC-siRNA to NC. The relative quantitative value of each sample was log2 transformed to conform the data for normal distribution. Quantified data between the two groups were evaluated using a two-tailed test. Differentially expressed proteins were filtrated based on the following criteria: fold change was equal to or greater than 1.2 and less than 0.83, and the P value was less than 0.05.

The protein ID was converted to UniProt ID, the corresponding Gene Ontology (GO) ID was obtained by searching the UniProt-GOA (www.http://www.ebi.ac.uk/GOA/) database, and GO was performed on differential protein annotations. For proteins that were not annotated, an algorithm software InterProScan (v.5.14-53.0, http://www.ebi.ac.uk/interpro/) was used to predict their GO functions. Kyoto Encyclopedia of Genes and Genomes (KEGG) annotation was realized using KAAS (v.2.0, http://www.genome.jp/kaas-bin/kaas_main). KEGG Mapper (v2.5, http://www.kegg.jp/kegg/mapper.html) was used to match the gene with the pathway in the database. The two-tailed Fisher's exact test was employed to evaluate the GO or KEGG pathway enrichment.

### Colony formation, Transwell, and wound-healing assays

Cells were seeded at 200 cells per well in a 6-well plate and cultured for 12 days during which DMEM was renewed every 4 days. The cells were then fixed with formaldehyde (ZhanWang Chemical, China) for 30 min and stained with crystal violet (Beyotime, China) for 10 min. An inverted microscope (Olympus, Japan) was used to count the number of clones with more than 50 cells at 100 × magnification.

Transwell chambers (Corning, USA) were coated with 10% Matrigel (BD, USA). Cells were first starved with serum-free DMEM for 12 h, and 1 × 10^5^ cells were then diluted with serum-free medium and seeded in the upper chamber. Complete medium was added to the lower chamber. After a 48-h incubation, cells remaining in the upper chamber were discarded. Chambers were fixed with formaldehyde for 30 min and stained with crystal violet for 10 min. Stained cells were photographed under a microscope with 200 × magnification.

We plated 3 × 10^5^ cells/well in a 6-well plate and allowed the cells to grow to a density of approximately 70%. A 10 ul pipette tip was used to draw a straight line in the center of each well. Scraped cells were washed off 3 times with phosphate buffered saline (PBS). Cells were then cultured and photographed at 0 h, 24 h, and 48 h under a microscope with 100 × magnification.

### Western blot analysis

A total of 40 μg of cell lysates was electrophoresed using 10% SDS-PAGE (Beyotime, China) and transferred to PVDF membranes (Millipore, USA). The membranes were blocked with 5% skimmed milk powder for 2 h and then incubated with the primary antibody at 4 °C overnight. The membranes were then incubated for 1.2 h at room temperature with the secondary antibody conjugated to a horseradish peroxidase-labeled anti-mouse IgG (1:20,000) (Zsbio, ZB-2305). Protein bands were detected using an ECL kit (Thermo, USA) according to the manufacturer’s protocol. Primary antibodies included mouse anti-PLA2G4A (1:500) (sc-376618, Santa Cruz, USA) and the internal control mouse anti-β-actin (1:1000) (TA-09, Zsbio, China).

### Statistical analysis

All experiments were performed in triplicate, and the data are expressed as mean ± standard error of the mean (SEM). Image J (National Institutes of Health, USA) was used to calculate cell numbers, scratch area, and band intensity. A two-tailed t-test was conducted using Graphpad Prism 7 software (Graphpad, USA), and the Fisher’s exact test was carried out using the Perl module (v.1.31, https://metacpan.org/pod/Text::NSP::Measures::2D::Fisher). P-values < 0.05 were considered statistically significant.

## Supplementary Information

Supplementary Information.

## Data Availability

The data used to support the findings of this study are available from the corresponding author upon reasonable request.

## References

[1] Thakkar JP (2014). Epidemiologic and molecular prognostic review of glioblastoma. Cancer Epidemiol. Biomark..

[2] Youssef Z (2019). New targeted gold nanorods for the treatment of glioblastoma by photodynamic therapy. J. Clin. Med..

[3] Polivka JJ (2017). Advances in experimental targeted therapy and immunotherapy for patients with glioblastoma multiforme. Anticancer Res..

[4] Ostrom QT (2018). CBTRUS statistical report: Primary brain and other central nervous system tumors diagnosed in the United States in 2011–2015. Neuro Oncol..

[5] Chen LL (2016). Linking long noncoding RNA localization and function. Trends Biochem. Sci..

[6] Panzitt K (2007). Characterization of HULC, a novel gene with striking up-regulation in hepatocellular carcinoma, as noncoding RNA. Gastroenterology.

[7] Xiong H (2017). LncRNA HULC promotes the growth of hepatocellular carcinoma cells via stabilizing COX-2 protein. Biochem. Biophys. Res. Commun..

[8] Liu T (2020). LncRNA HULC promotes the progression of gastric cancer by regulating miR-9-5p/MYH9 axis. Biomed. Pharmacother..

[9] Dong Y (2019). Long non-coding RNA HULC interacts with miR-613 to regulate colon cancer growth and metastasis through targeting RTKN. Biomed. Pharmacother..

[10] Chu P, Xu LN, Su HY (2019). HULC functions as an oncogene in ovarian carcinoma cells by negatively modulating miR-125a-3p. J. Physiol. Biochem..

[11] Yan H (2016). High expression of long noncoding RNA HULC is a poor predictor of prognosis and regulates cell proliferation in glioma. Oncol Targets Ther..

[12] Zhu Y (2016). HULC long noncoding RNA silencing suppresses angiogenesis by regulating ESM-1 via the PI3K/Akt/mTOR signaling pathway in human gliomas. Oncotarget.

[13] Islam F, Gopalan V, Lam AK (2020). Mass spectrometry for biomarkers discovery in esophageal squamous cell carcinoma. Methods Mol. Biol..

[14] Zhao Q (2020). Proteogenomics uncovers a vast repertoire of shared tumor-specific antigens in ovarian cancer. Cancer Immunol. Res..

[15] Deb B (2020). Bioinformatics analysis of global proteomic and phosphoproteomic data sets revealed activation of NEK2 and AURKA in cancers. Biomolecules.

[16] Li HL (2019). Assessing the utility of multiplexed liquid chromatography-mass spectrometry for gluten detection in Australian breakfast food products. Molecules.

[17] Shao Y (2020). Long non-coding RNA PVT1 regulates glioma proliferation, invasion, and aerobic glycolysis via miR-140–5p. Eur. Rev. Med. Pharmacol. Sci..

[18] Huang W (2020). LncRNA GAS5-AS1 inhibits glioma proliferation, migration, and invasion via miR-106b-5p/TUSC2 axis. Hum. Cell..

[19] Zhang Q (2020). Comprehensive analysis of the long noncoding RNA expression profile and construction of the lncRNA-mRNA co-expression network in colorectal cancer. Cancer Biol. Ther..

[20] Cao YP (2020). Long non-coding RNA in bladder cancer. Clin. Chim. Acta..

[21] Kore RA, Abraham EC (2014). Inflammatory cytokines, interleukin-1 beta and tumor necrosis factor-alpha, upregulated in glioblastoma multiforme, raise the levels ofCRYAB in exosomes secreted by U373 glioma cells. Biochem. Biophys. Res. Commun..

[22] Wu R (2019). HMGB1 contributes to SASH1 methylation to attenuate astrocyte adhesion. Cell. Death. Dis..

[23] Cheng PJ (2011). Differential proteomics analysis of amniotic fluid in pregnancies of increased nuchal translucency with normal karyotype. Prenat. Diagn..

[24] Riedl J, Ay C (2019). Venous thromboembolism in brain tumors: Risk factors, molecular mechanisms, and clinical challenges. Semin. Thromb. Hemost..

[25] Mathur R (2020). MGMT promoter methylation level in newly diagnosed low-grade glioma is a predictor of hypermutation at recurrence. Neuro Oncol..

[26] Zhang SC (2017). m6A demethylase ALKBH5 maintains tumorigenicity of glioblastoma stem-like cells by sustaining FOXM1 expression and cell proliferation program. Cancer Cell.

[27] Niu Y (2019). RNA N6-methyladenosine demethylase FTO promotes breast tumor progression through inhibiting BNIP3. Mol. Cancer..

[28] Leslie CC (2015). Cytosolic phospholipase A_2_: Physiological function and role in disease. J. Lipid Res..

[29] Zhao HY (2018). MiR-543 promotes migration, invasion and epithelial-mesenchymal transition of esophageal cancer cells by targeting phospholipase A2 group IVA. Cell Physiol. Biochem..

[30] Bai HS (2020). PLA2G4A is a potential biomarker predicting shorter overall survival in patients with non-M3/NPM1 wildtype acute myeloid leukemia. DNA Cell Biol..

[31] Tunset HM (2019). Cytosolic phospholipase A2 alpha regulates TLR signaling and migration in metastatic 4T1 cells. Int. J. Mol. Sci..

[32] Pang X (2019). cPLA2a correlates with metastasis and poor prognosis of osteosarcoma by facilitating epithelial-mesenchymal transition. Pathol. Res. Pract..

[33] Hanahan D, Weinberg RA (2011). Hallmarks of cancer: The next generation. Cell.

[34] Tsuji S (2019). Temozolomide has anti-tumor effects through the phosphorylation of cPLA2 on glioblastoma cells. Brain Res..

