# Supplementary material for: Quantitative proteomics analysis of glioblastoma cell lines after lncRNA HULC silencing
Source: Sci Rep. 2021 Jun 15;11:12587. doi: 10.1038/s41598-021-92089-z (PMC8206103; doi:10.1038/s41598-021-92089-z)
Supplement: Supplementary file 1 — Supplementary Information. [file 41598_2021_92089_MOESM1_ESM.pdf]

Quantitative proteomics analysis of glioblastoma cell lines after lncRNA HULC silencing

Shan Ye, Jing Wu, Yiran Wang, Yuchen Hu, Tiantian Yin, Jie He

A

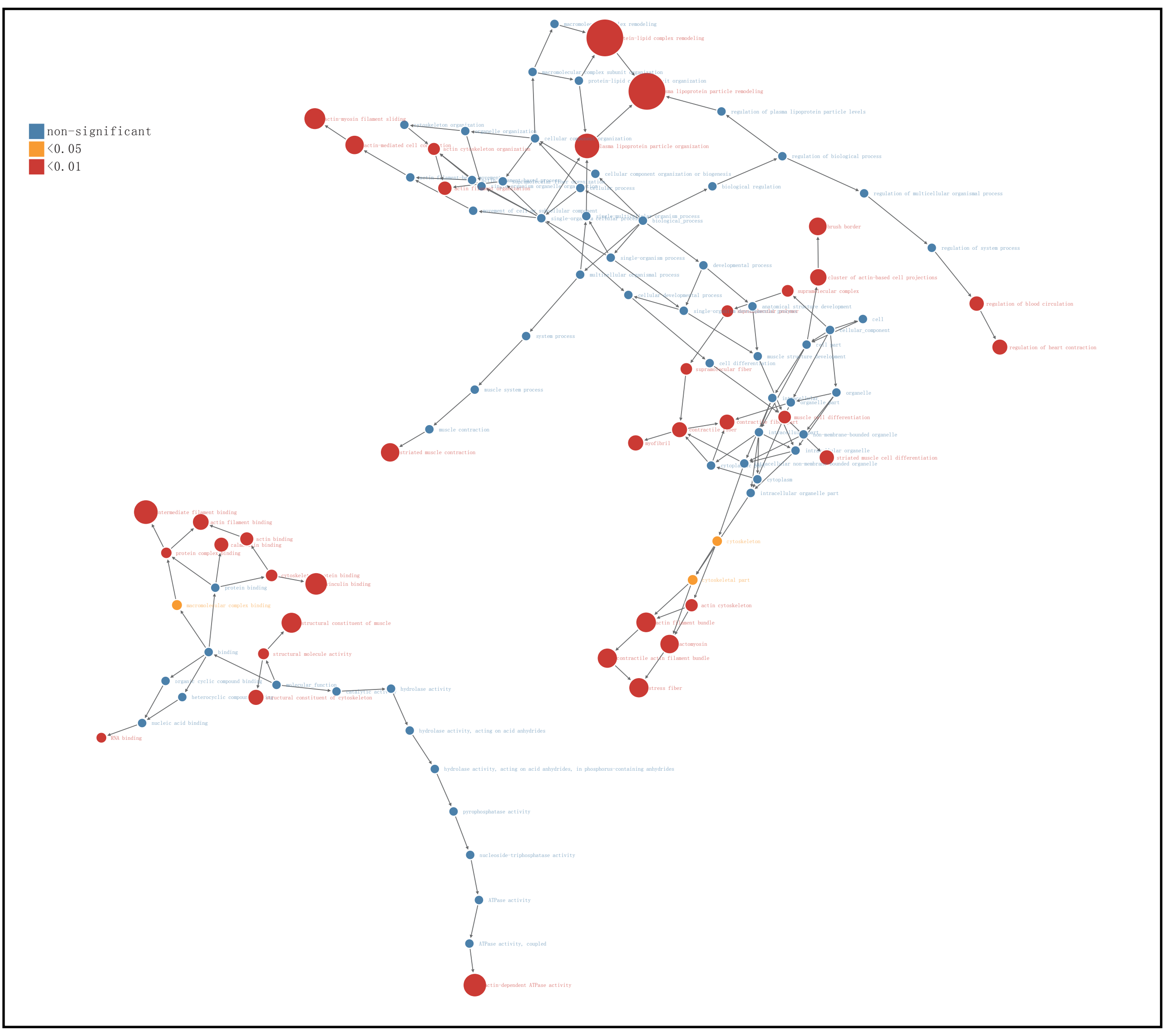

B

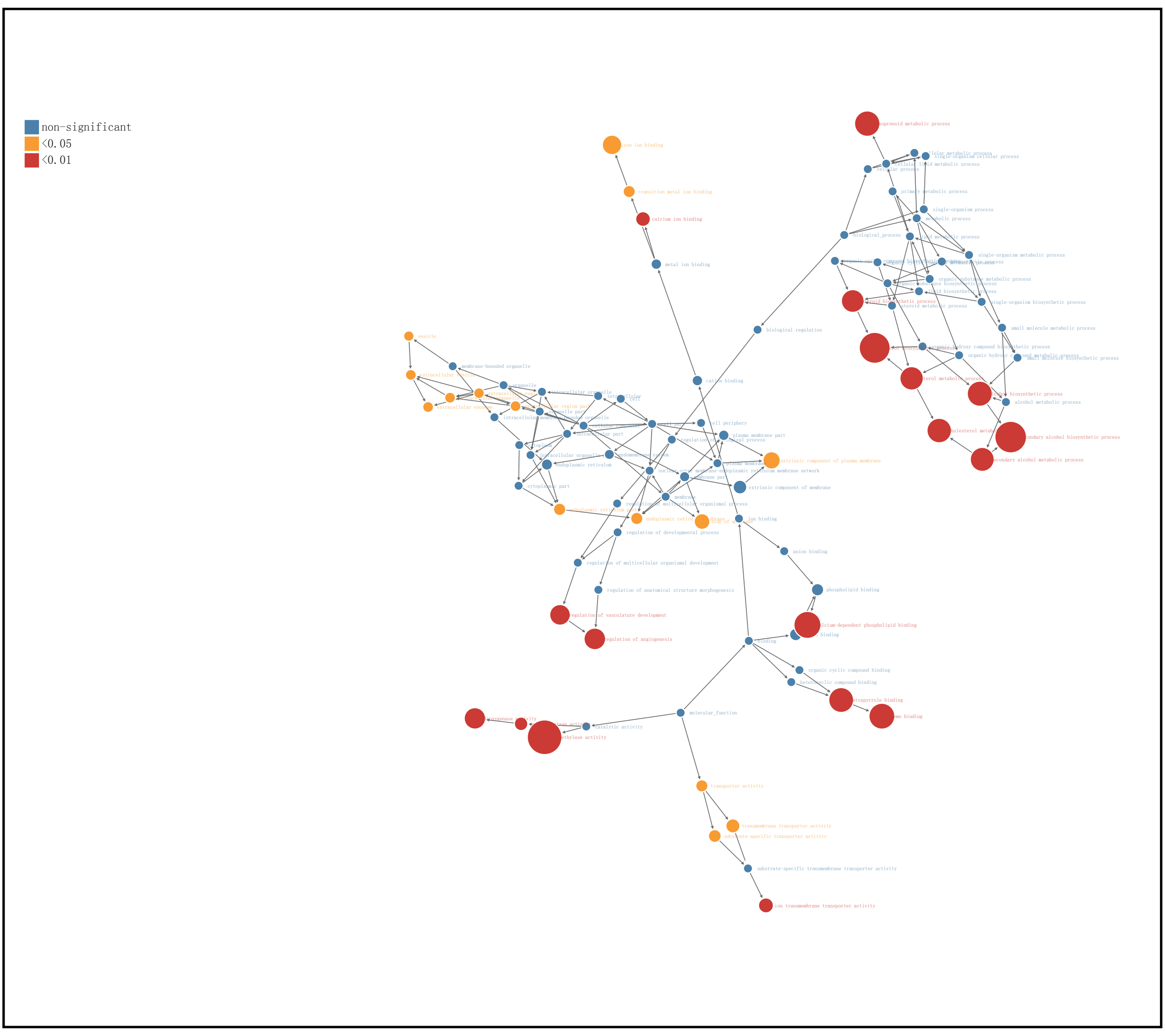

Supplementary Figure S1. Directed acyclic graph for GO enrichment of dysregulated proteins.
(A) The graph presents the hierarchical relationships of up-regulated GO functions.
(B) GO enrichment of down-regulated proteins focused on deeper levels.

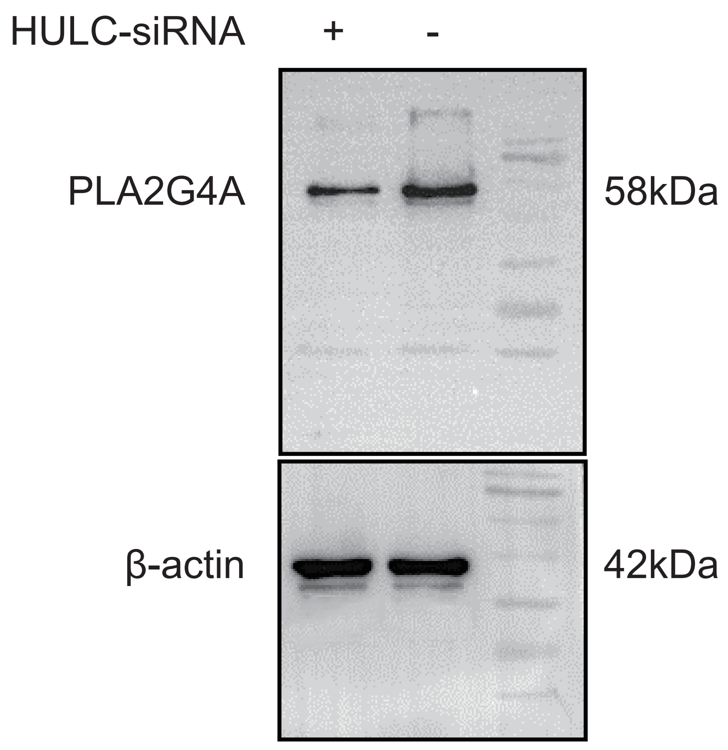

Supplementary Figure S2. Original version of protein abundance of PLA2G4A determined by Western blot analysis ($P < 0.001$).
